# A Quantitative Characterization of Nucleoplasmin/Histone Complexes Reveals Chaperone Versatility

**DOI:** 10.1038/srep32114

**Published:** 2016-08-25

**Authors:** Noelia Fernández-Rivero, Aitor Franco, Adrian Velázquez-Campoy, Edurne Alonso, Arturo Muga, Adelina Prado

**Affiliations:** 1 Department of Biochemistry and Molecular Biology, Faculty of Science and Technology, University of the Basque Country (UPV/EHU), P.O. Box 644, E-48080 Bilbao, Spain; 2 Biofisika Institute (CSIC, UPV/EHU), P. O. Box 644, 48080 Bilbao, Spain; 3 Institute of Biocomputation and Physics of Complex Systems (BIFI), Joint Unit IQFR-CSIC-BIFI, Universidad de Zaragoza, C/Mariano Esquillor, Zaragoza 50018, Spain; 4 Department of Biochemistry and Molecular and Cell Biology, University of Zaragoza, Zaragoza 50009, Spain; 5 Instituto de Investigaciones Sanitarias de Aragón (IIS-A), Zaragoza 50009, Spain; 6 Fundación ARAID, Government of Aragón, Zaragoza 50018, Spain

## Abstract

Nucleoplasmin (NP) is an abundant histone chaperone in vertebrate oocytes and embryos involved in storing and releasing maternal histones to establish and maintain the zygotic epigenome. NP has been considered a H2A–H2B histone chaperone, and recently it has been shown that it can also interact with H3-H4. However, its interaction with different types of histones has not been quantitatively studied so far. We show here that NP binds H2A–H2B, H3-H4 and linker histones with K_d_ values in the subnanomolar range, forming different complexes. Post-translational modifications of NP regulate exposure of the polyGlu tract at the disordered distal face of the protein and induce an increase in chaperone affinity for all histones. The relative affinity of NP for H2A–H2B and linker histones and the fact that they interact with the distal face of the chaperone could explain their competition for chaperone binding, a relevant process in NP-mediated sperm chromatin remodelling during fertilization. Our data show that NP binds H3-H4 tetramers in a nucleosomal conformation and dimers, transferring them to DNA to form disomes and tetrasomes. This finding might be relevant to elucidate the role of NP in chromatin disassembly and assembly during replication and transcription.

*Xenopus laevis* early embryogenesis is a process characterized by rapid cell division and transcriptional quiescence that depends on parental stored proteins, including histones^1^. Histone chaperones bind these basic ligands to store or escort them to their final destinations^2^, and to modulate the post-translational modifications that regulate their chromatin remodelling activity^3^,^4^,^5^,^6^.

Nucleoplasmin (NPM2, here called NP), a member of the nucleophosmin/nucleoplasmin family of histone chaperones^7^, is involved in H2A–H2B storage and chromatin remodelling^8^. NP forms stable homopentamers comprised of 22 kDa subunits that fold into a two domain structure (Fig. 1A): an eight-stranded β-barrel N-terminal core domain (residues 16–120) with a jelly roll topology (Fig. 1B)^7^,^9^,^10^,^11^,^12^, and the C-terminus tail that adopts a disordered conformation^13^. NP contains three acidic tracts, A1, A2 (or polyGlu) and A3, the last two being part of the C-terminal intrinsically disordered domain (Fig. 1A) that builds the so-called distal face of the protein pentamer (Fig. 1C). This domain also contains the positively charged, bipartite nuclear localization sequence (NLS) (Fig. 1A)^7^,^14^,^15^. Post-translational modifications (PTMs), in particular phosphorylation, of NP activates its chromatin decondensation activity, enhancing its ability to remove linker histones from DNA^16^,^17^, and increases its affinity for H2A–H2B and H5^18^,^19^. NP co-immunoprecipitates not only with H2A–H2B but also with H3-H4 in *Xenopus* oocyte and egg extracts, suggesting that it can interact with both histones^20^. Electron microscopy (EM) analysis of full-length, native NP from *Xenopus laevis* eggs (eNP), isolated and in complexes with H2A–H2B, H3-H4 or histone octamers shows the highly acidic distal face of the chaperone contacting all types of histones^19^,^20^. Interestingly, the interaction of eNP with H2A–H2B, H3-H4 or the histone octamer results in different complexes. As seen by EM and analytical ultracentrifugation, one eNP pentamer can bind 5 H2A–H2B dimers at the distal face, whereas a larger ellipsoidal complex is formed with the H3-H4 tetramer either alone or as part of the histone octamer, in which the basic ligands are wrapped by the distal faces of two NP pentamers^20^. Furthermore, the NP distal face also binds linker histones H1 and H5 and linker-related, sperm-specific basic proteins (SSBP)^17^. Thus, NP shares with other histone chaperones the capacity to bind several histone ligands, a property that is probably related to the distinct biological processes these proteins are involved in^5^,^21^,^22^,^23^. For instance, NP has been proposed to play a role in histone storage in the oocyte, decondensation of sperm chromatin after fertilization, replication licensing, and nucleosome assembly in early embryonic cells^15^,^24^. NP could interact with different histone partners in these processes, being able to distinguish them as suggested by the distinct complexes they form.

In this study, we have characterized the affinity of NP isolated from *X. laevis* oocytes (oNP) or eggs (eNP) and of full-length and truncated forms of recombinant NP (rNP) (Fig. 1A) for linker (H1 and H5) histones, H2A–H2B dimers, and H3-H4 dimers and tetramers. It should be noted that the affinity of NP isolated from natural sources for its basic ligands and the interaction of this chaperone with H3-H4 have not been yet characterized. We aim to understand how PTMs modulate NP affinity for the different types of histones and the protein regions that stabilize the different chaperone/histone complexes. Our data show that natural NPs (eNP and oNP) carrying several PTMs bind core histones with apparent affinities in the subnanomolar range, whereas the affinity of non-phosphorylated recombinant protein (rNP) for core histone dimers (H3-H4/H2A–H2B) is 65–120-fold lower. The mechanism by which PTMs modulate NP affinity for histones most likely involves exposure of the polyGlu (A2) tract, as deletion of the last 50 residues of the C-terminal domain restores the affinity of the recombinant protein to values similar to those of natural NPs. We also find that NP binds both association states of H3-H4, and that the chaperone-bound tetramer adopts a nucleosomal conformation that can be transferred to DNA. We suggest that formation of stereospecific NP/histone complexes could be related to the role of NP in chromatin disassembly and assembly, which could mediate recovery of nucleosomes that must be transiently disrupted to allow passage of DNA and RNA polymerases

## Results

### Regulation of the interaction of NP with H2A–H2B dimers

**Figure 1:**
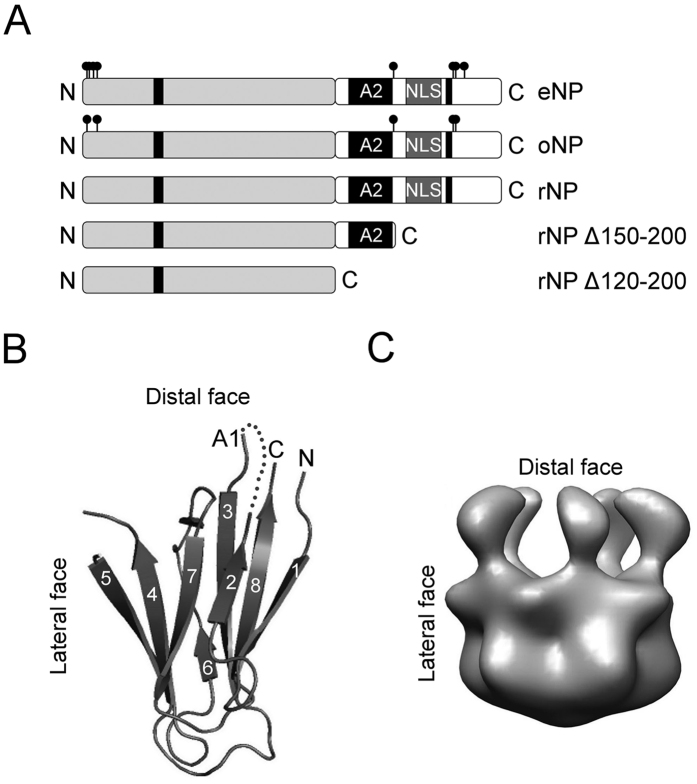
Structural properties of Nucleoplasmin. (**A**) Schematic representation of the primary structure of native egg and oocyte NP, full length recombinant NP (rNP) and the two deletion mutants rNPΔ150–200 and rNPΔ120–200 used in this study. The core (residues 1–120; light gray) and tail (residues 121–200; white) domains are also shown. Phosphorylation sites (circles) and the location of the three acidic tracts (A1, A2, A3; black boxes) and the NLS (dark grey box) are highlighted. (**B**) Crystal structure of the N-terminal core domain of one NP monomer (amino acids 16–120). The location of the A1 acidic tract (dotted line), and of the distal, and lateral protein faces is indicated (PDB 1K5J). (**C**) Electron microscopy reconstruction of eNP. Side view of the final volume of the three-dimensional reconstruction of eNP^19^.

**Figure 2:**
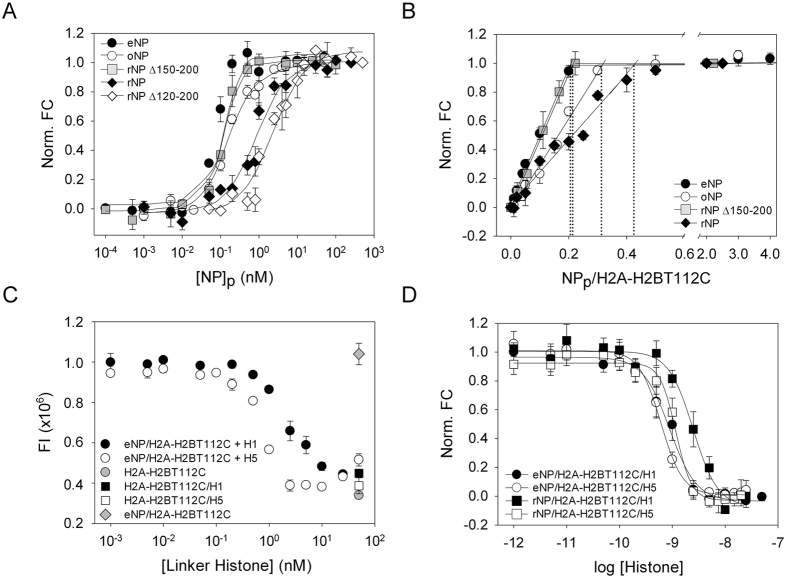
Phosphorylation and exposure of the polyGlu tract modulates NP affinity for H2A–H2B and linker histones. (**A**) Normalized fluorescence intensity change (Eq. 1) of H2A–H2BT112C-Alexa 488 at increasing concentration of eNP (filled circles), oNP (empty circles), rNPΔ150–200 (gray squares), rNP (filled diamonds), and rNPΔ120–200 (empty diamonds). In this particular experiment histone concentration was 1 nM and NP concentration is given for the protein pentamer. Values of the mean ± SEM from three independent experiments are shown. The K_d_ values obtained from fitting the experimental data to *eqs.* 2 and 3 are included in Table 1. (**B**) Determination of the saturation stoichiometry of the NP/H2A–H2B complexes. Normalized change in the fluorescence intensity (Eq. 1) as a function of the NP pentamer/H2A–H2BT112C-Alexa molar ratio. The intersection of the linear phase with the plateau gives the molar ratio at which pentameric eNP (filled circles), oNP (empty circles), rNPΔ150–200 (gray squares), and rNP (filled diamonds) are saturated with H2A–H2BT112C. (**C**) Fluorescence competition assays in which complexes of eNP (0.1 nM)/H2A–H2B-Alexa 488 (1 nM) are competed with increasing concentrations of unlabelled H1 (filled circles) or H5 (empty circles). Controls of the fluorescence intensity of H2A–H2BT112C-Alexa 488 alone (gray circles), and in the presence of 0.3 μM H1 (filled squares), 0.3 μM H5 (empty squares) or 0.1 nM eNP (gray diamonds). **D**) Normalized fluorescence change (Eq. 1) of eNP/ (circles) or rNP/H2A–H2BT112C-Alexa (squares) complexes titrated with increasing concentrations of unlabelled H1 (filled symbols) or H5 (empty symbols). NP and H2A–H2BT112C-Alexa concentrations were 0.1 and 1 nM, respectively for the eNP/H2A–H2B complex, and 0.5 and 5 nM for the rNP/H2A–H2B complex. Experimental data were fitted to *eqs.* 6 and 7. Values of the mean ± SEM from three independent experiments are shown. The apparent K_d_ values are shown in Table 1.

Although the role of NP as a H2A–H2B histone chaperone is widely recognized, the effect of PTMs on histone binding has not been quantitatively characterized yet. Special care was taken to preserve the phosphorylation state of both natural NP species during the purification procedure. The different phosphorylation of rNP, oNP and eNP was confirmed by their distinct migration in SDS- and Native-PAGE^13^,^20^. Using a fluorescence-based method^25^, we determine herein the affinity of natural and key mutant NPs for H2A–H2B dimers. The fluorescence intensity of H2A–H2BT112C-Alexa 488 increased in the presence of NP at 0.15 M NaCl but not at 2M salt, conditions that precluded the interaction (Supplementary Fig. 1A). Fluorescence enhancement arises most likely from a change in the chemical environment of the fluorophore due to a conformational change of the ligand upon complex formation. This interpretation is supported by the similar increase in the fluorescence intensity of H2A–H2B in 2 M NaCl, which was not sensitive to NP (Supplementary Fig. 1A). The apparent affinity of H2A–H2BT112C for different NP variants was estimated by measuring the fluorescence intensity at increasing NP concentration (Fig. 2A; eq. 1). It was previously shown that labelling H2A–H2BT112C with Alexa 488 did not affect assembly of the NP/core histone complexes^20^. Experimental data were fitted to the ligand-depleted binding model described in Materials and Methods (*Eqs.* 2 and 3), which gave the K_d_ values included in Table 1. Comparison of these values indicates that the apparent affinity of H2A–H2BT112C-Alexa dimer for both natural NPs (oNP and eNP) was similar and in the subnanomolar range, whereas that for rNP was 65–120 times lower. This finding demonstrates that PTMs, particularly phosphorylation, regulate the affinity of NP for H2A–H2B, as it was suggested using phosphomimetic mutants^18^. To analyse the contribution of the core and tail domains to chaperone affinity, we used two truncated chaperone variants that lack the last 50 residues (rNPΔ150–200) or the whole C-terminal domain (rNPΔ120–200) (Fig. 1A). Deletion of the last 50 residues at the C-terminus, which contains the positively charged NLS, recovered the affinity of the recombinant protein to levels comparable to those of the phosphorylated natural proteins, whereas the affinity of the NP core (rNPΔ120–200) was only 1.5-fold lower than that of rNP (Fig. 2A and Table 1). These data point out that exposure of the polyGlu tract upon elimination of the last 50 residues of the tail domain regulates the affinity of NP for H2A–H2B. The apparent saturation stoichiometry of the eNP/ and rNPΔ150–200/H2A–H2B complexes was 5, in agreement with previous studies^19^, whereas for oNP and rNP it dropped to 3 and 2, respectively (Fig. 2B). This suggests that the binding capacity of NP can also be regulated by two different, but most likely related -see discussion-, mechanisms: phosphorylation of the native proteins and exposure of the polyGlu tract in the recombinant species.

### Linker histones outcompete H2A–H2B for NP binding: consequences for nucleosome assembly

It has been previously shown that eNP remodels the chromatin from somatic nuclei and *Xenopus* sperm by extracting H1/H5 and SSBP, respectively^3^,^15^,^17^,^24^. This reflects the ability of NP to interact with these three types of linker histones. Indeed, we show using an *in vitro* assay with *Xenopus* spermatic DNA incubated with preformed NP/H2A–H2B complexes, that natural NPs and rNPΔ150–200 completely dissociated the SSBP from DNA (Supplementary Fig. 2A,B) and simultaneously mediated nucleosome assembly (Supplementary Fig. 2C). In contrast, neither rNPΔ120–200 nor rNP significantly removed SSBPs or assembled nucleosomes. To explore whether the chaperone affinity for linker histones and H2A–H2B could explain this finding, we performed competition experiments in which NP/H2A–H2BT112C-Alexa 488 complexes were titrated with unlabelled H1 or H5 (Fig. 2C,D). Addition of either H5 or H1 induced a decrease in the fluorescence intensity of the preformed complex, indicating that linker histones outcompeted H2A–H2B for NP binding. The apparent K_d_ values obtained using *eqs.* 6 and 7 for the interaction of H1 and H5 with eNP were 0.07 and 0.03 nM, respectively, values similar (for H1) or slightly lower (around 2-fold for H5) than those estimated for H2A–H2B (Fig. 2D and Table 1). The apparent affinities of both linker histones for eNP were 24-fold higher than for rNP (K_d_ = 0.7 and 1.7 nM), in accordance with ITC data showing a similar increase in the affinity of a phosphomimetic recombinant protein variant for H5^18^. These data support the proposals that PTMs modulate the affinity of NP for linker histones and that binding of SSBPs to the eNP/H2A–H2B complex, which occurs during sperm chromatin decondensation, could be coupled to the transfer of eNP-bound H2A–H2B to the tetrasome during nucleosome assembly^15^,^26^. They thus indicate that NP-mediated histone exchange regulates the chaperone remodelling activity, which in turn depends on PTMs.

### NP binds both H3-H4 dimers and tetramers with high affinity

**Figure 3:**
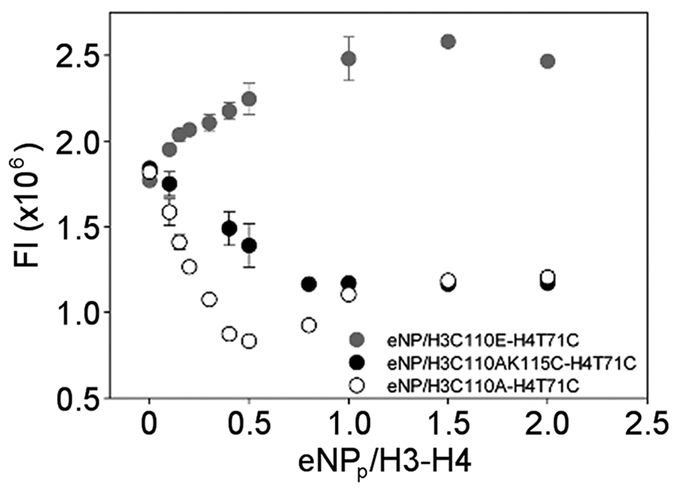
Nucleoplasmin binds H3C110A-H4 dimers and tetramers, forming different types of complexes. Fluorescence intensity change as a function of the eNP/H3-H4 molar ratio for the different H3-H4 variants: H3C110A-H4T71C-Alexa 488 (empty circles), H3C110E-H4T71C-Alexa 488 (gray circles), and cross-linked H3C110AK115C-H4T71C-Alexa 488 (filled circles). Histone concentration was kept constant at 10 nM. Experimental data are means ± SEM from three independent experiments.

Although the existence of NP/H3-H4 complexes in extracts of *X. laevis* eggs and oocytes has been recently demonstrated^20^, neither the affinity nor the stoichiometry of these complexes has been quantitatively determined. This would be essential to understand the role of NP as a H3-H4 histone chaperone. To quantitatively characterize the interaction, we carried out fluorescence titration experiments. As aforementioned for H2A–H2B, labelling of H3C110A-H4T71C with Alexa 488 did not affect complex formation with NP^20^. In contrast to what was obtained for H2A–H2B, the interaction of H3-H4 with eNP induced a biphasic fluorescence intensity change (Fig. 3). At NP/H3-H4 molar ratios lower than 1/2 the fluorescence decreased with increasing NP concentrations and partially recovered at higher molar ratios up to 1, where it reached a constant value. This behaviour might be due to the interaction of NP with H3-H4 tetramers or dimers at NP/histone molar ratios lower or higher than 1/2, respectively. As previously published^20^, Native-PAGE analysis of the eNP/H3-H4 samples showed the presence of low and high molecular weight bands, the latter being evident at molar ratios lower than 1/2 (Supplementary Fig. 3A), which reveals the existence of at least two major distinct assemblies. To prove the above hypothesis, we measured the interaction of NP with H3C110E-H4T71C, a predominantly dimeric histone variant^27^, and with cross-linked H3C110AK115C-H4T71C tetramers^28^,^29^. In accordance with this hypothesis, the fluorescence of dimeric H3-H4 increased in the presence of NP whereas that of cross-linked H3-H4 tetramers decreased (Fig. 3), explaining therefore the interaction of natural H3-H4 with the chaperone.

**Figure 4:**
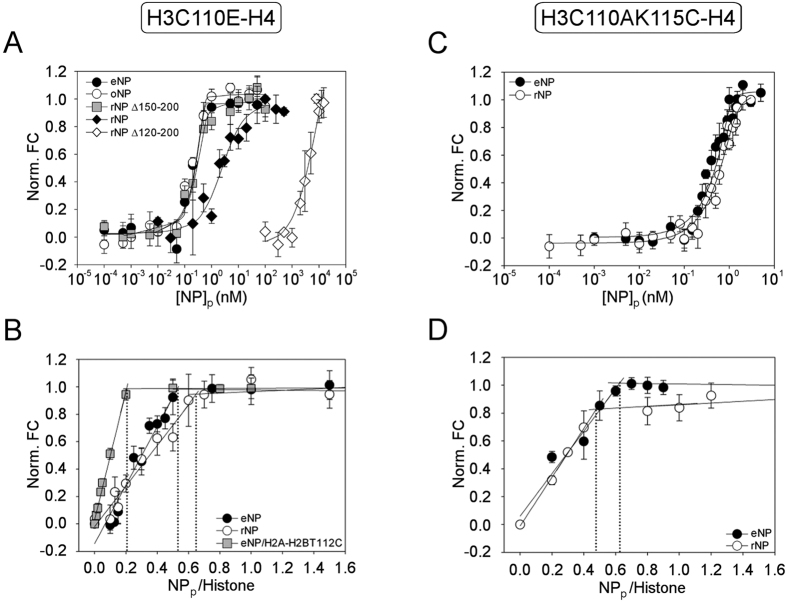
NP affinity for H3-H4 dimers and cross-linked tetramers. (**A**) Titration of H3C110E-H4T71C-Alexa 488 with different NP variants. Normalized fluorescence intensity change (Eq. 1) of H3C110E-H4T71C-Alexa 488 as a function of eNP (filled circles), oNP (empty circles), rNPΔ150–200 (gray squares), rNP (filled diamonds), or rNPΔ120–200 (empty diamonds) concentration. In this particular experiment histone concentration was 1 nM and NP concentration is given for the pentamer. (**B**) Normalized fluorescence intensity change (Eq. 1) of H3C110E-H4T71C-Alexa 488 as a function of the NP pentamer/histone molar ratio. The saturation stoichiometry of samples containing eNP (filled circles) or rNP (empty circles) is estimated from the intersection of the linear phase with the plateau. Histone concentration was kept constant and 10-fold higher than the apparent K_d_ estimated for each complex. Data corresponding to the eNP/H2A–H2BT71C-Alexa 488 complexes (gray squares) are also shown for the sake of comparison. (**C**) Normalized fluorescence intensity change (Eq. 1) of cross-linked H3C110AK115C-H4T71C-Alexa 488 (2 nM) as a function of eNP (filled circles) or rNP (empty circles) concentration. (**D**) Saturation stoichiometry of complexes formed by cross-linked H3C110AK115C-H4T71C and eNP (filled circles) or rNP (empty circles). Other details as in B. Binding data (**A,C**) were fitted to the ligand-depleted model described in Materials and Methods using *eqs.* 4 and 5. The estimated K_d_ values are shown in Table 1. Data in A-D are means ± SEM from at least three independent experiments.

To estimate the apparent affinity of the two association states of H3-H4 for NP, dimeric H3C110E-H4T71C and the cross-linked, tetrameric variant, both labelled with Alexa 488, were titrated with natural and recombinant NPs. The fluorescence intensity of the dimeric mutant increased upon addition of NP at 150 mM NaCl and slightly decreased at 2 M salt (Supplementary Fig. 1B). Fitting of the experimental data with a ligand-depleted binding model that considers two potential binding sites in NP for H3-H4 dimers (Fig. 4A; *eqs.* 4 and 5) yielded the apparent K_d_ values included in Table 1. As shown above for H2A–H2B, the apparent affinity of eNP for dimeric H3-H4 is 2-fold and 65-fold higher than those of oNP and rNP, respectively (Table 1), thus suggesting that PTMs also modulate the affinity of NP for dimeric H3-H4. The similarity between the affinities of NP for the two types of core histones extends to the inhibitory effect of the last 50 residues on the interaction (Fig. 4A and Table 1). Further deletion of the polyGlu tract in the rNPΔ120–200 mutant caused an abrupt drop in affinity, as seen by a five orders of magnitude increase in the apparent K_d_ value (Fig. 4A and Table 1). Interestingly, this polyGlu-associated affinity change is significantly higher for H3-H4 than for H2A–H2B dimers. The K_d_ values, 16 × 10^3^ and 11 nM for H3-H4 and H2A–H2B dimers, indicate that the NP core binds H3-H4 with a markedly lower affinity and thus, that the tail domain is essential to stabilize this complex (Table 1). The interaction of NP with both histone dimers also differs in the stoichiometry of the complexes. Whereas eNP could bind 5 H2A–H2B dimers, it was saturated with 2 H3-H4 dimers (Fig. 4B). In contrast to H2A–H2B, the maximum number of H3-H4 molecules that NP can bind was not sensitive to PTMs. These data suggest that NP displays a comparable affinity for dimers of both core histones *in vitro*, albeit it forms different complexes with them^19^,^20^.

The interaction of the chaperone with tetrameric H3-H4 was explored by titrating cross-linked H3-H4 tetramers with eNP and rNP (Fig. 4C). The normalized fluorescence intensity change (Eq. 1) was analysed with a ligand-depleted binding model that considers a saturation stoichiometry of one cross-linked tetramer per NP pentamer (see below). The apparent affinity of the H3-H4 tetramer for eNP was only slightly higher, around 2-fold, than for rNP (Fig. 4C and Table 1), and both NP pentamers can accommodate under saturation conditions one cross-linked tetramer (Fig. 4D). The apparent molecular mass of this complex derived from Native-PAGE, around 440 kDa, was similar for samples composed of cross-linked H3-H4 tetramers or low NP/native H3-H4 molar ratios (Supplementary Fig. 3A,B). As suggested by EM, these high MW complexes are formed by two NP pentamers and four H3-H4 dimers, at least two forming a tetramer, placed at the centre of the ellipsoidal particle^20^. In contrast, the low MW would contain one NP pentamer and two H3C110E-H4T71C dimers (Fig. 4B). Another difference observed between these complexes is their ionic-dependent stability. Whereas complex formation with dimeric H2A–H2B or H3-H4 was hampered at 2 M NaCl (Supplementary Fig. 1A,B), a similar decrease in fluorescence intensity was detected regardless of the ionic strength of the medium when eNP is incubated with cross-linked H3-H4 tetramers (Supplementary Fig. 1C). This suggests that electrostatic contacts stabilize the complex of NP with dimeric histones, whereas hydrophobic and/or hydrogen bonding interactions might explain the persistence of the eNP/H3-H4 tetramer complexes at high ionic strength. This interpretation would also explain the higher affinity of eNP, but not rNP, for dimeric H3-H4. Cross-linking of the H3-H4 tetramer might induce conformational rearrangements in the histone that could expose hydrophobic surfaces responsible for its lower affinity for the highly charged, phosphorylated eNP.

### Association state and conformation of NP-bound H3-H4

**Figure 5:**
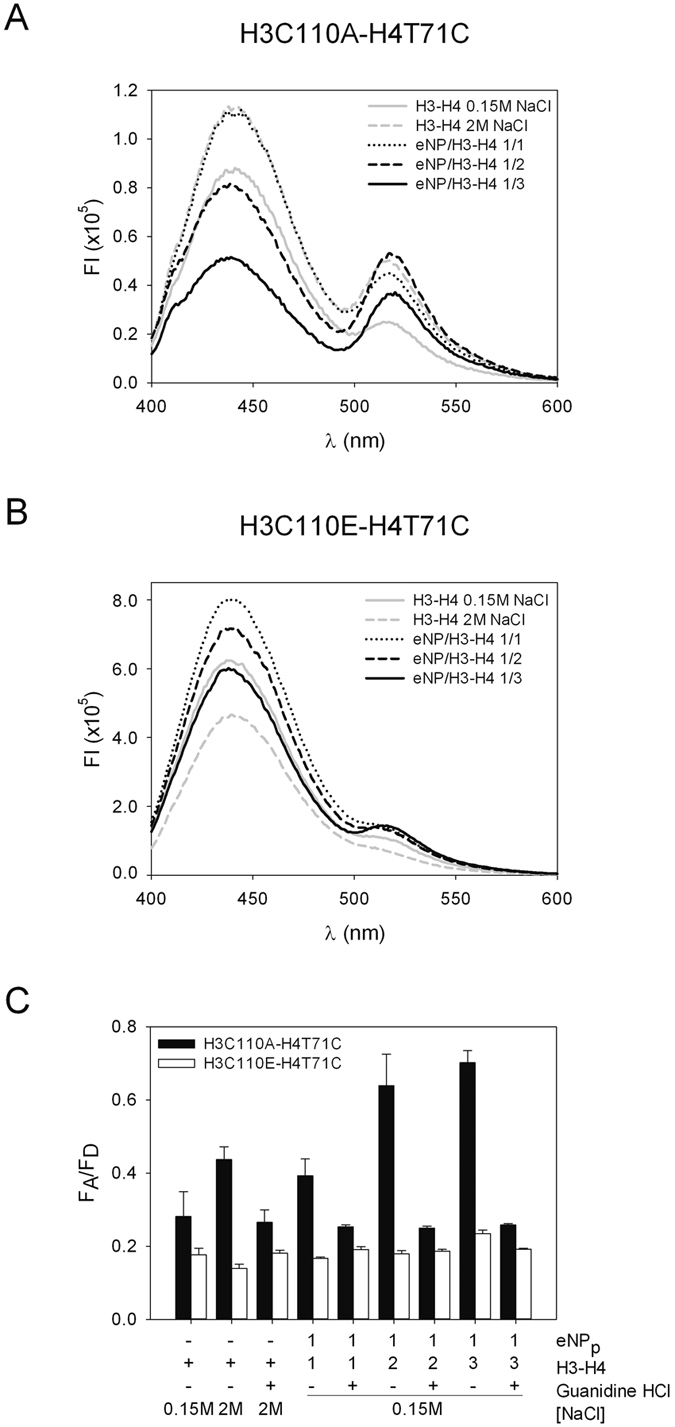
NP stabilizes a tetrameric H3-H4 conformation. FRET analysis of eNP/H3-H4T71C complexes formed upon incubation of H3-H4T71C labelled with Alexa 350 (0.5 μM) or 488 (0.5 μM) with eNP. (**A**) Emission spectra of H3C110A-H4T71C-Alexa in 0.15 M NaCl (gray solid line), 2 M NaCl (gray dashed line), and of eNP/H3C110A-H4T71C complexes obtained in 0.15 M NaCl at the following molar ratios: 1/1 (black dotted line), 1/2 (black dashed line), and 1/3 (black solid line). Excitation wavelength was 359 nm. (**B**) Emission spectra of H3C110E-H4T71C-Alexa. Other details as in A. (**C**) Comparison of fluorescence energy transfer of the samples shown in A and B, expressed as the ratio of the emission at 519 and 442 nm. The values for the same histone mixtures in 0.15 M NaCl, 2 M NaCl, and for the eNP/H3-H4 complexes in 0.15 M NaCl and 3 M guanidine HCl are also shown.

The association state of eNP-bound H3-H4 was analysed by FRET using H3-H4T71C labelled with Alexa 350, which would act as donor, or Alexa 488, which would be the acceptor molecule provided that the distance between both probes is shorter than R_o_, 50 Å for this pair (Fig. 5). FRET will be only observed when the H3-H3′ dimer interaction surface places the donor and acceptor probes close enough, a situation that only occurs for the tetrameric histone conformation^30^. FRET would cause a decrease and increase of the donor and acceptor fluorescence intensities, respectively, as it was observed for H3C110A-H4T71C in the presence of 2 M NaCl (Fig. 5A) and more markedly when bound to the chaperone at eNP pentamer/H3-H4 dimer molar ratios lower than 1/2, or in others words when NP is saturated and the high MW complex appears (Fig. 5A,C). These spectral changes were not detected for dimeric H3C110E-H4T71C under the same experimental conditions (Fig. 5B,C). These data suggest that H3C110A-H4T71C-Alexa bound to the chaperone or at 2 M NaCl adopts a compact structure compatible with a tetramer. In stark contrast, the F_A_/F_D_ values obtained for dimeric H3C110E-H4T71C-Alexa indicate that this mutant does not form a stable tetramer neither in 2 M salt nor bound to eNP (Fig. 5B,C).

**Figure 6:**
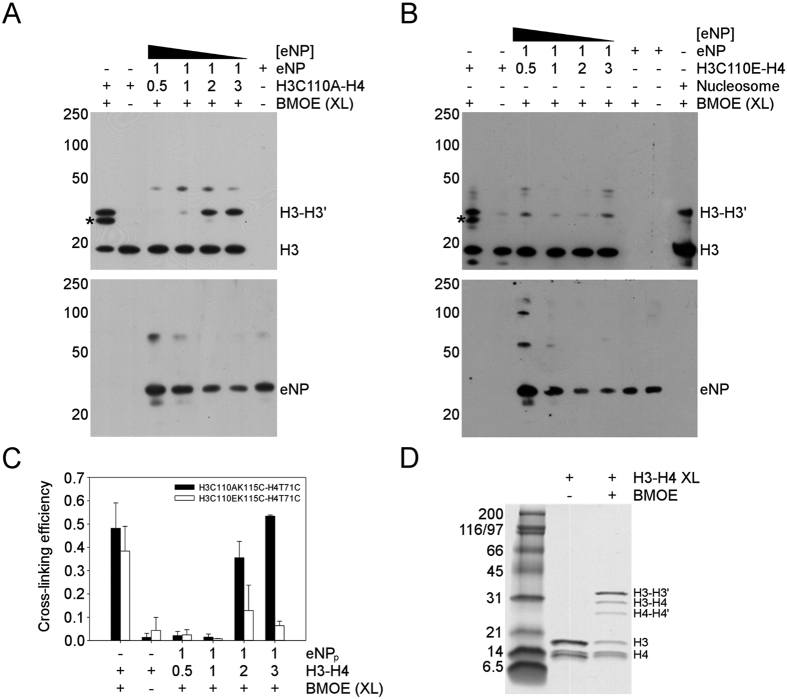
Directed sulfhydryl reactive cross-linking suggests that NP stabilizes a nucleosomal H3-H4 conformation. (**A**) Cross-linking of eNP/H3C110AK115C-H4T71C complexes revealed by electrophoretic separation of the reaction components and western blotting with anti-H3 (upper panel) and anti-NP (lower panel). (**B**) Same as in A for eNP/H3C110EK115C-H4T71C complexes. Cross-linked bands of eNP or free histones in 0.15 M NaCl (**A,B**) and histones in the nucleosome (**B**) are also shown as controls. (**C**) Cross-linking efficiency of the samples analysed in **A** and **B** estimated as described in the Materials and Methods section. (**D**) Identification of the different H3 and H4 cross-linked adducts by SDS-PAGE.

To further characterize the association state of NP-bound H3-H4, we performed cross-linking experiments using the H3K115C-H4T71C variants that harbour a cysteine, C115, close to the H3-H3′ interacting surface and the cross-linker BMOE. This cross-linker covalently attaches cysteine residues of two dimers, forming a H3-H3′ adduct if 115C-115C´ residues are closer than 8 Å, a situation that occurs when they form a tetramer^28^. Cross-linking of eNP/H3C110A-H4 complexes obtained at different chaperone/histone molar ratios revealed that a H3-H3′ adduct was formed when the eNP pentamer binds two H3-H4 dimers, yielding the large MW complex (Fig. 6A). At higher NP/H3-H4 molar ratios, when the probability of two H3-H4 dimers bound to the same NP pentamer is low, cross-linking was not detected (Fig. 6A). The assignment of this band to H3-H3′ has been made considering the cross-linking of isolated histones at 0.15 M NaCl and 1 μM dimer concentration (Fig. 6A). Cross-linked samples were analysed by denaturing NuPAGE, followed by western blot with anti-H3 (Fig. 6A upper panel) and anti-NP (Fig. 6A lower panel), and by SDS-PAGE to assign the different adducts (Fig. 6D). The H3-H4′ adduct observed when the histones were free in solution was not detected when they formed part of the histone octamer in reconstituted nucleosomes (Fig. 6B) or when they were complexed with NP (Fig. 6A,B). The absence of the H3-H4′ adduct in the nucleosome and chaperone complexes also suggests that the conformation of H3-H4 in both complexes might be similar^31^, and less heterogeneous than that of free histones in solution. Estimation of the relative amount of the H3-H3′ adduct in the eNP/H3-H4 complexes by densitometry revealed that cross-linking requires binding of two histone dimers to one NP pentamer (Fig. 6C). The same experiment was performed with the H3C110E-H4T71C variant, whose association equilibrium is shifted towards the dimer (Fig. 6B). In this case, eNP-bound histones failed to significantly form cross-linked species (Fig. 6B upper panel and 6C), indicating that their formation relies on the ability of the histone dimers to tetramerize. Summarizing, FRET and cross-linking experiments indicate that NP-bound H3-H4 adopts a conformation similar to that found in the nucleosome^31^.

### NP-mediated tetrasome assembly

**Figure 7:**
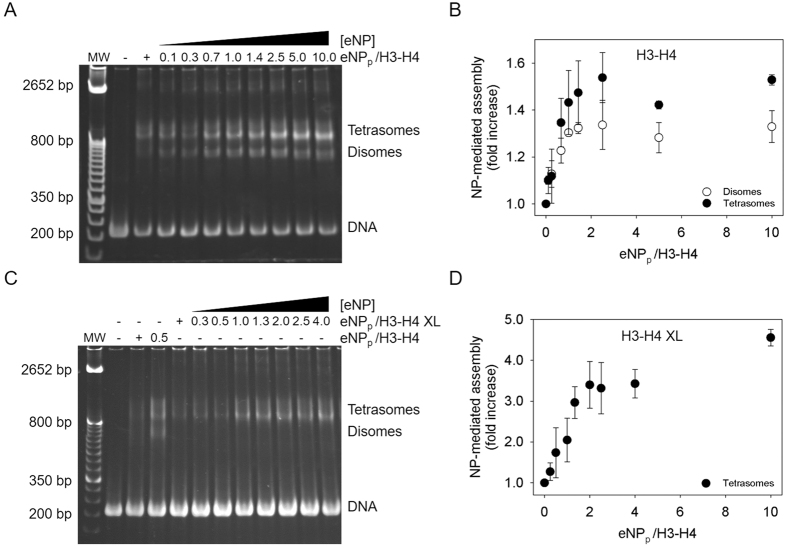
NP facilitates transfer of H3-H4 tetramers to DNA. (**A,C**) EMSA studies of disome and tetrasome formation by native H3-H4 or cross-linked H3C110AK115C-H4T71C. 0.8 μM histones were incubated with increasing eNP concentrations for 1 h before addition of 0.4 μM DNA. After 2 h at room temperature and 30 min at 42 °C, samples were analysed by Native-PAGE. The position of the disome, tetrasome and free DNA bands is indicated. (**B,D**) Quantification of the disome and tetrasome bands shown in A and C by densitometry. The NP-mediated disome and tetrasome assembly was estimated as the ratio of the intensity of the disome or tetrasome bands in the presence and absence of eNP. Means ± SEM from at least three independent experiments.

We next wanted to know whether NP could transfer H3-H4 to DNA to form tetrasomes and disomes. To explore this potential ability, we incubated DNA with eNP/H3-H4 complexes obtained at different chaperone/histone molar ratios, and followed formation of tetrasomes and disomes (Fig. 7). eNP mediated transfer of both association states of natural H3-H4 to DNA, as seen by the intensity of the bands assigned to disomes and tetrasomes^32^ (Fig. 7A,B). Chaperone-mediated deposit of cross-linked H3-H4 tetramers to DNA further supports this ability (Fig. 7C,D).

## Discussion

Histone chaperones can be grouped in two classes according to their interaction with histones. The first one includes chaperones that *in vitro* bind most of the chromatin related histones with similar affinities, thus being generic; although *in vivo* they could interact preferentially with a particular type of histone. An example of this class would be Nap1 that has been considered a H2A–H2B histone chaperone *in vivo*^33^ and interacts *in vitro* with H1, H2A–H2B dimers and H3-H4 dimers and tetramers^25^,^31^,^34^,^35^. Members of the second class bind both core histones with different affinities, as found for CAF-1 that displays an affinity for H3-H4 20-fold higher than for H2A–H2B^36^. The opposite has been described for FACT, which *in vitro* binds H2A–H2B 20 times better than H3-H4, and it can interact simultaneously with H2A–H2B and H3-H4 dimers during transcription^37^. NP has been considered a H2A–H2B histone chaperone, although it has also been demonstrated that it can interact with linker histones H5 and H1^17^,^24^, SSBPs^17^, both association states of H3-H4^8^,^20^,^38^ and with the histone octamer^20^,^39^. Furthermore, the specificity of a chaperone for a given ligand may be regulated *in vivo* by PTMs or through direct competition among different basic partners. A quantitative characterization of the affinity of the different NP species used in this study for the distinct types of histones could help to understand the chaperone activity of NP, and to evaluate the role of PTMs and domain architecture in its chromatin remodelling activity.

Among the post-translational modifications that NP undergoes, which include acetylation^40^, arginine methylation and glutamylation^41^, phosphorylation has been proposed to regulate the affinity of NP for histones. It has been shown that hyperphosphorylation allows NP to compete with DNA for histone linker binding during sperm decondensation^3^,^17^,^40^,^41^. Studies using phosphomimetic variants have also suggested that phosphorylation could modulate the affinity of NP for linker and H2A–H2B histones^18^, although a quantitative estimation of the affinity of natural hyperphosphorylated NP for the different histones has not been attempted yet. The characterization of these interactions is biologically relevant, as they have been found to occur in extracts of *Xenopus* eggs and oocytes^20^. Our data establish that eNP and oNP interact with linker and core histones with apparent K_d_ values in the subnanomolar range. They also show that the affinity of the chaperone for these basic ligands is regulated by PTMs, as demonstrated by the reduction in the affinity of recombinant NP as compared with natural NPs, and by exposure of the polyGlu tract, which induce a similar increase in the affinity of recombinant rNPΔ150–200 for core histones. NP phosphorylation has been extensively characterized^13^,^41^,^42^. It has been proposed that phosphorylation or deletion of the last 50 residues of the C-terminal domain might induce a similar exposure of the polyGlu tract. Phosphorylation of the full-length protein, and especially of residues flanking the positively charged nuclear localization sequence, could disrupt the interaction between this C-terminal protein region and the polyGlu tract that controls the accessibility of this negatively charged segment^43^. An increased accessibility of this negatively charged protein region could explain the expansion of the hyperphosphorylated eNP pentamer due to electrostatic repulsion among protomers within the pentamer, its ability to decondense chromatin, as compared with rNP^17^, and the recently described enhanced glutamylation of a protein variant carrying phosphomimetic mutations^41^. Exposure of the polyGlu tract upon deletion of the region containing the NLS in rNPΔ150–200 promotes the expected recovery of the chaperone affinity for both types of histones to values characteristic of the natural phosphorylated proteins. Although additional effects from phosphorylation of the N-terminal region of the protein and other PTMs cannot be ruled out, our data suggest that phosphorylation-mediated changes in polyGlu accessibility regulates the affinity of NP for core and linker histones. Further deletion of the 30 residues of the C-terminal domain that contain the polyGlu (A2) tract in rNPΔ120–200 reduces two and five orders of magnitude the affinity of NP for H2A–H2B and H3-H4, respectively. The slightly higher charge per amino acid estimated for H3-H4 (0.16 *vs.* 0.14 for H2A–H2B)^44^,^45^ might explain, at least partially, this difference. The balance between ionic and hydrophobic interactions, as derived from the electrostatic dependence of NP binding to these histones, might also influence the distinct structures of the complexes. The ellipsoidal eNP/H3-H4 particle is made of two NP pentamers and 4 H3-H4 dimers^20^, while in the cup-like eNP/H2A–H2B complex each C-terminal domain of NP interacts with a histone dimer^19^. This means that only one or several C-terminal phosphorylated domains are required to bind a H2A–H2B or H3-H4 dimer, respectively. The lower apparent saturation stoichiometry observed for rNP/H2A–H2B complexes suggests that reduction of the negative charge at the non-phosphorylated, distal protein face imposes that more than one protomer are required to neutralize and bind a histone dimer. In contrast, the stoichiometry of the NP/H3-H4 complexes is not sensitive to PTMs, suggesting that the concerted action of several non-phosphorylated protomers retains the capacity to bind two H3-H4 dimers, albeit with a markedly lower affinity.

The affinity of linker histone for NP is 17 times higher than that estimated for linker DNA^46^, thus quantitatively supporting the ability of NP to remove linker histones and induce chromatin decondensation^3^,^17^,^24^,^26^,^47^. eNP, oNP and rNPΔ150–200 in complex with H2A–H2B are able *in vitro* to decondense *Xenopus* sperm chromatin and assemble nucleosomes. This finding strongly suggests that removal of linker histones by the NP/H2A–H2B complex is coupled to core histone deposition into tetrasomes to assemble nucleosomes, a process that takes place during *Xenopus* egg fertilization^15^,^26^,^47^. The use of the same disordered NP distal face to bind both histones would favour histone exchange.

The interaction of a NP pentamer with one H3-H4 dimer results in formation of a low molecular mass complex similar to those previously described with H2A–H2B under the same experimental conditions^19^. However, when NP binds two H3-H4 dimers or a cross-linked tetramer, a football-shaped particle is obtained^20^. Our results demonstrate that in this complex, eNP-bound H3-H4 adopts a tetrameric conformation similar to that found in the nucleosome. This finding could be relevant in the recycling and *de novo* deposition of histone dimers and tetramers during replication and transcription. A key question is whether recycling of parental histones involves the direct transfer of a H3-H4 tetramer or its splitting into two dimers that can be reassembled as a tetramer^48^. The exchange rates of H2A–H2B are at least 20-fold higher than those of H3-H4 in transcribed regions^49^,^50^, suggesting that H3 and H4 have their own recycling mechanism during nucleosome remodelling^51^. It is also known that nucleosome assembly on DNA during replication and transcription involves redeposition of old H3-H4 tetramers without splitting them into dimers^52^,^53^,^54^. These observations are difficult to reconcile with the chaperone-induced tetramer splitting that has been described during assembly and disassembly of chromatin. Recent studies have found that persistence of the parental tetramers might be due to different modes of interaction between histone chaperones and their cargo. They show that Nap1 and Vps75, which also bind all four core histones with nanomolar affinity *in vitro*^25^,^55^,^56^,^57^, stabilize the tetrasomal configuration of H3-H4^58^. An additional chaperone that binds both association states of H3-H4 is Spt2, which targets a H3-H4 tetramer by interacting with its periphery without interfering with the tetramerization interface^59^.

Our data add NP to the list of histone chaperones that can bind *in vitro* both association states of H3-H4 with high affinity, forming however complexes with different structural properties. In the complex with the tetramer, the C-terminal tails of NP create an internal cage^20^ where histones are stabilized in a conformation similar to that found in the nucleosome. This mode of binding ensures that once removed from DNA by remodelling factors, H3-H4 tetramers could be directly transferred to NP, which would stabilize them avoiding splitting and mediate their deposition into newly synthesized DNA. This interpretation is reinforced by the ability of NP to transfer cross-linked H3-H4 tetramers to DNA. It is also tempting to speculate that the different modes of binding of H2A–H2B and H3-H4 at the surface of the chaperone and buried within the NP cage, respectively, might be related with their different exchange rates. The accessibility of the confined NP-bound H3-H4 tetramer could be regulated by additional proteins and/or DNA. The finding that NP is even able to bind and assist in the deposition of H3-H4 dimers onto DNA forming disomes *in vitro*, as found for Asf1^32^, also points to a possible role of NP as a reservoir of soluble H3-H4 and in the incorporation of newly synthesized H3-H4 into DNA. The versatility of NP to transfer H3-H4 dimers and tetramers and its relatively high affinity for both association states of these core histones suggest that NP could regulate the histone species that are available for direct deposition onto DNA during transcription and replication.

In summary, data presented here show that NP binds *in vitro* both linker and core histones with high affinity (apparent K_d_ in the subnanomolar range), forming complexes with different stoichiometry and architecture. The affinity of the chaperone for its basic ligands is modulated by the accessibility of the acidic tract at the intrinsically disorder distal face, which in turn is regulated by phosphorylation. We demonstrate that the use of the same structural region of the chaperone to interact with all types of histones facilitates histone exchange, an essential process for nucleosome dynamics. Linker histones and H2A–H2B competition for NP binding might favour the synchronized sperm chromatin decondensation and release of the maternal H2A–H2B dimer bound to NP to assemble the male pronucleus. Our data also point to the ability of NP to form stereospecific complexes with histones that could be related to the efficient deposition of histone components during nucleosome assembly. Binding of H3-H4 tetramers in a nucleosomal conformation could facilitate eNP-mediated transfer of the tetramer during replication and transcription. The flexibility of the disordered distal region could also aid in the structural adaptation of NP to form different complexes with these ligands.

## Materials and Methods

### Protein purification

Oocyte NP (oNP) and egg NP (eNP) from *X. laevis* were purified as previously published^13^. Recombinant NPs (full-length and two truncated variants that lack the last 50 -rNPΔ150–200- or 80 -rNPΔ120–200- residues) were expressed and purified as described^13^. Natural source H2A–H2B, and H3-H4 were obtained from chicken erythrocyte chromatin upon elution from a hydroxyapatite column^60^ and kept at 4 °C in 2 M NaCl until use. H1 and H5 were obtained from chicken erythrocyte chromatin^61^. Recombinant histones, including mutants (H2BT112C, H3C110E, H3C110A, H4T71C) and (H3C110AK115C, H3C110EK115C) were expressed, purified and reconstituted according to previously published protocols^25^,^58^. The cross-linked tetramer was prepared as described^28^ at a dimer (H3C110AK115C-H4T71C)/bis-maleimidoethane (BMOE) (Thermo Fisher Scientific) molar ratio of 1/1. The cross-linking efficiency, as seen by SDS-PAGE, was 80% and the cross-linked tetramer was used directly in the NP-tetramer interaction assay. NP concentration was determined by the bicinchoninic acid assay (Sigma), and, unless otherwise stated, is given for its pentameric form. The concentration of natural source histones was determined by absorbance at 230 nm, using extinction coefficients of ε_230_ = 4.3 cm^2^mg^−1^ for the H2A–H2B dimer and ε_230_ = 4.1 cm^2^mg^−1^ for the H3-H4 dimer, all in water. H1 and H5 were quantified according to^61^. The concentration of recombinant histones was estimated by densitometry using known amounts of H3-H4 or H2A–H2B as standards. Histone concentration is given for the dimeric species (H3-H4 and H2A–H2B). Molar ratios are expressed as NP pentamer/H3-H4 or H2A–H2B dimer or linker histone monomer.

### Antibodies

Polyclonal anti-NP was obtained from Abyntek Biopharma S.L. (Bilbao, Spain). Anti-H3 (ab 1791) was from Abcam (Cambridge, England) and Rabbit Pierce Goat anti-Rabbit IgG (H+L) peroxidase conjugated from Thermo Fisher Scientific.

### Preparation of DNA

The DNA fragment of 207 bp containing the *Lytechinus variegatus* rDNA sequence was prepared by restriction enzyme digestion, HhaI (New England Biolabs) and RsaI (Sigma), with subsequent gel purification from the plasmid p5S207-12, followed by PCR amplification using the (F: 5′ act tgc atg gga gac cgc ctg gga ata c/R: 5′ act aac cga gcc cta tgc tgc ttg act t) primers and DNA gel purification.

### Analysis of the Disome and Tetrasome complexes

#### Salt dialysis assembly

Nucleosomes, containing H3C110AK115C-H4 tetramers, and tetrasomes were assembled by salt dialysis as described^62^. 207 base pair DNA, comprising the 5S rRNA gen of *Lytechinus variegatus*, was mixed at a DNA/H3-H4 tetramer and octamer molar ratio of 1/1 in 2 M NaCl, 20 mM Tris-HCl, pH 7.5, and 0.2 mM EDTA. The final DNA concentration was 0.1–0.2 mg/ml. Samples were subjected to stepwise salt dialysis to reach a NaCl concentration of 0.15 M.

#### NP-mediated assembly

NP/H3-H4 complexes assembled at different NP/H3-H4 molar ratios were mixed with DNA (207 bp; 0.4 μM final concentration) in 150 mM NaCl, 20 mM Tris-HCl, pH 7.6, 0.2 mM EDTA, 1 mM DTT, 10% glycerol, 0.2 mg/ml BSA. The DNA/H3-H4 tetramer molar ratio was kept at 1/1. Disome and tetrasome assembly was analysed by 6% Native-PAGE electrophoresis. Gels were stained with SYBR Green EMSA (Life Technologies, Oregon), and the relative amount of tetrasomes and disomes was estimated by densitometry using the Quantity One Program (BioRad). The NP-mediated assembly was estimated as X_i_/X_o_; where X_i_ is the intensity of the disome or tetrasome band obtained in the presence of NP, and X_0_ that measured in the absence of the chaperone.

### Fluorescence spectroscopy assays

#### Protein labelling

H2BT112C and H4T71C were labelled with Alexa 488 (Invitrogen) at a protein/fluorescent probe molar ratio of 1/5, in 20 mM Tris-HCl, pH 7.2, 1 mM TCEP (Tris (2-carboxyethyl) phosphine), 6 M guanidine hydrochloride, and incubated overnight at 4 °C as indicated^63^. Labelled histones were mixed with H2A, H3C110A, H3C110E or H3C110AK115C to reconstitute complexes^25^ that were purified by size exclusion chromatography with a Superdex 200 16/60 column (GE Healthcare). Labelling efficiency was 75% and 40% for H2BT112C and H4T71C, respectively. The concentration of labelled histone dimers was estimated by densitometry using known amounts of H2A–H2B and H3-H4 as standards.

#### Fluorescence titration assay

Fluorescence at 519 nm was measured in a Horiba Jobin Yvon Fluorolog-3 Spectrofluorometer, after excitation at 495 nm, using excitation and emission slits of 8 nm. The affinity of each NP species for the different types of histones was estimated by titrating different concentrations (0.1–10 nM) of labelled histones with increasing chaperone concentrations. The normalized fluorescence change (Norm FC) was determined once the fluorescence signal had reached equilibrium^25^, and plotted as a function of protein concentration. Experiments with H2A–H2BT112C, H3C110A-H4T71C and H3C110E-H4T71C were performed in 150 mM NaCl, 20 mM Tris-HCl, pH 7.6, 1 mM DTT, 0.1 mg/ml BSA, and with cross-linked H3C110AK115C-H4T71C, in 150 mM NaCl, 20 mM Tris-HCl, pH 7.6, 1 mM DTT, 1% glycerol, 0.05% Tween 20. The normalized fluorescence change was calculated according to the following equation:


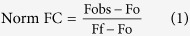


where F_obs_ is the experimental fluorescence; F_0_ is the fluorescence in the absence of NP; and F_f_ is the fluorescence intensity at saturating NP concentrations.

Binding stoichiometries were estimated by titrating a concentration of labelled histones 10 times higher than the apparent K_d_ of complex formation with the corresponding NP. Fluorescence intensity changes until NP is saturated with histones, and the molar ratio at the inflection point gives the number of NP and histone molecules in the saturated complex.

### Data analysis

To analyse the fluorescence titrations, we have considered a ligand-depleted binding model in which NP has 5 potential binding sites for H2A–H2B dimers, as indicated previously^19^ and suggested by data presented herein. Mass conservation coupled to chemical equilibrium leads to the following equation:


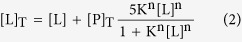


where [P]_T_ and [L]_T_ are the total concentrations of NP (in a pentameric basis) and histone dimer, respectively, [L] is the concentration of free histone dimer, K is the association constant for the NP/histone dimer interaction, and n is the Hill coefficient. Solving this equation allows calculating complex concentration and the fluorescence signal, F, as a function of reactant concentrations:


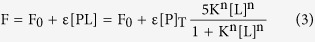


where F_0_ is the initial fluorescence value (no NP added), and ε is the change in fluorescence emission of the complex. Because the fluorescence signal along the experiment has been normalized, F_0_ and ε[P]_T_ must be close to 0 and 1. Non-linear regression analysis was performed implementing the model in Origin 7 (OriginLab).

To analyse the fluorescence titrations for H3-H4, a ligand-depleted binding model considering 2 binding sites in NP for H3-H4 dimers has been considered, as estimated from binding experiments. Mass conservation coupled to chemical equilibrium leads to the following equation:


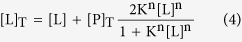


As described above, solving that equation allows calculating the concentration of complex and the fluorescence signal, F, as a function of reactant concentrations:


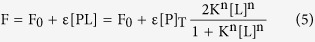


Similarly, the assays with cross-linked H3-H4 were analysed considering a 1/1 NP pentamer/histone tetramer binding stoichiometry at saturation.

### Competition assays

NP/H2A–H2B-Alexa 488 complex was competed with unlabelled H1 or H5. The experimental conditions were the following: labelled H2A–H2B concentration was kept constant and similar to the apparent K_d_ for NP/H2A–H2B complex formation; NP concentration was 10 times lower than the apparent K_d_, and the fluorescence of the sample was measured at increasing concentrations of unlabelled H1 or H5^46^. Normalized fluorescence data were fitted to the following dose-response equation to estimate EC_50_.


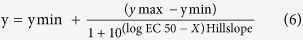


EC_50_ is the linker histone concentration that reduces the amount of NP/H2A–H2B-Alexa complex signal by 50% and depends on the labelled H2A–H2B concentration and the competitor’s binding affinity as described^46^. Once the apparent K_d_ for the NP/H2A–H2B complex is known, that for the NP/linker histone complex is calculated by the Cheng-Prusoff equation^64^.


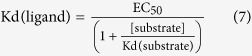


K_d_ (ligand) is the apparent K_d_ for NP/linker histone complex formation; [substrate] is the labelled H2A–H2B concentration; and K_d_ (substrate) the apparent K_d_ for the NP/H2A–H2B complex. Fluorescence emission at 519 nm was measured after excitation at 495 nm with both slits at 8 nm in a Horiba Jobin Yvon Fluorolog-3 Spectrofluorometer.

### FRET experiments

The association state of H3-H4 bound to NP was analysed using two different populations of H4T71C labelled with Alexa Fluor 488 (acceptor) or Alexa Fluor 350 (donor). Reconstituted labelled H3-H4 histones were extensively dialyzed in 150 mM NaCl, 10 mM Tris-HCl, pH 7.5, 1 mM EDTA, 5 mM β-Mercaptoethanol. Equimolar mixtures of H3-H4T71C-Alexa 488 and H3-H4T71C-Alexa 350 were incubated with different NP concentrations in 150 mM NaCl, 20 mM Tris-HCl, pH 7.6, 1 mM DTT, 0.1 mg/ml BSA. Histone concentration was kept at 1 μM and NP/histone complexes and controls were incubated for 1 h at room temperature. Longer incubation times did not change the FRET signal. Emission spectra were recorded between 400 and 600 nm, using an excitation wavelength of 359 nm and setting both the excitation and emission slits at 2 nm, in a Horiba Jobin Ybon Fluorolog-3 Spectrofluorometer.

### Cross-linking experiments

H3C110AK115C-H4T71C or H3C110EK115C-H4T71C/chaperone complexes were formed at 25 °C in 150 mM NaCl, 20 mM Tris-HCl, pH 7.5, 1mM EDTA, after incubating different eNP concentrations with a constant histone concentration (1 μM dimer) during 1 h. BMOE was added at a cross-linker/histone dimer molar ratio of 1/1 and the reaction was allowed to proceed for 60 s at room temperature. Afterwards, 1 mM DTT was added to quench the reaction^28^,^58^. Quenching proceed for 5 minutes before SDS loading buffer was added and samples were boiled at 100 °C and resolved by NuPAGE (4–12% acrylamide) (Life technologies, Carlsbad, CA, USA). NP and histone bands were analysed by western blot and the antibodies were detected with the chemiluminiscence method (Super Signal West Pico Chemiluminescent substrate, Thermo Fisher Scientific). Quantification was done by densitometry using the Quantity One Program (BioRad).

## Additional Information

**How to cite this article**: Fernández-Rivero, N. *et al*. A Quantitative Characterization Of Nucleoplasmin/Histone Complexes Reveals Chaperone Versatility. *Sci. Rep.* **6**, 32114; doi: 10.1038/srep32114 (2016).

## Supplementary Material

Supplementary Information

## Figures and Tables

**Table 1:** Apparent dissociation constants (K_d_) estimated for different NP/histone complexes.

	K_d_ (nM)				
	eNP	oNP	rNP	rNP Δ150–200	rNP Δ120–200
H2A–H2BT112C	0.06 ± 0.03	0.110 ± 0.007	7.0 ± 0.8	0.12 ± 0.04	11 ± 2
H3C110E-H4T71C (dimer)	0.050 ± 0.004	0.09 ± 0.07	3 ± 2	0.05 ± 0.03	16000 ± 2000
H3C110AK115C/H4T71C(Cross-linked tetramer)	1.10 ± 0.09		2.1 ± 0.2		
H1	0.07 ± 0.03		1.70 ± 0.60		
H5	0.030 ± 0.006		0.7 ± 0.2		

Different concentrations of labelled H2A–H2BT112C, H3C110E-H4T71C or cross-linked H3C110AK115C-H4T71C (0.1–10 nM) were titrated with eNP, oNP, rNP and with the truncated variants rNPΔ150–200 and rNPΔ120–200. Competition experiments were carried out titrating NP/H2A-H2B complexes with different histone linker concentrations. The apparent K_d_ values were obtained using the Cheng-Prusoff equation (Eq. 7) or the ligand depleted binding models described in the Materials and Methods section (*Eqs.* 3 and 5). The Hill coefficient (n) was 1 for all samples, except for binding of cross-linked H3-H4 tetramers to eNP (n = 0.88) or rNP (n = 0.73). Data are the average of at least two experimental measurements at each histone concentration.

## References

[b1] Almouzni G. & Wolffe A. P. Constraints on transcriptional activator function contribute to transcriptional quiescence during early Xenopus embryogenesis. EMBO J 14, 1752–1765 (1995). 7737126 10.1002/j.1460-2075.1995.tb07164.x PMC398268

[b2] Gurard-Levin Z. A., Quivy J.-P. & Almouzni G. Histone Chaperones: Assisting Histone Traffic and Nucleosome Dynamics. Annu Rev Biochem 83, 487–517, doi: 10.1146/annurev-biochem-060713-035536 (2014). 24905786

[b3] Leno G. H., Mills A. D., Philpott A. & Laskey R. A. Hyperphosphorylation of nucleoplasmin facilitates Xenopus sperm decondensation at fertilization. J Biol Chem 271, 7253–7256 (1996). 8631735 10.1074/jbc.271.13.7253

[b4] Leno G. H. Cell-free systems to study chromatin remodeling. Methods Cell Biol 53, 497–515 (1998). 9348522 10.1016/s0091-679x(08)60892-4

[b5] Tyler J. K. Chromatin assembly. Cooperation between histone chaperones and ATP-dependent nucleosome remodeling machines. Eur J Biochem/FEBS 269, 2268–2274 (2002). 10.1046/j.1432-1033.2002.02890.x 11985607

[b6] Hondele M. & Ladurner A. G. The chaperone-histone partnership: for the greater good of histone traffic and chromatin plasticity. Curr Opin Struct Biol 21, 698–708, doi: 10.1016/j.sbi.2011.10.003 (2011). 22054910

[b7] Eirin-Lopez J. M., Frehlick L. J. & Ausio J. Long-term evolution and functional diversification in the members of the nucleophosmin/nucleoplasmin family of nuclear chaperones. Genetics 173, 1835–1850, doi: 10.1534/genetics.106.058990 (2006). 16751661 PMC1569712

[b8] Dilworth S. M., Black S. J. & Laskey R. A. Two complexes that contain histones are required for nucleosome assembly *in vitro*: role of nucleoplasmin and N1 in Xenopus egg extracts. Cell 51, 1009–1018 (1987). 3690659 10.1016/0092-8674(87)90587-3

[b9] Dutta S. . The crystal structure of nucleoplasmin-core: implications for histone binding and nucleosome assembly. Mol Cell 8, 841–853 (2001). 11684019 10.1016/s1097-2765(01)00354-9

[b10] Namboodiri V. M., Dutta S., Akey I. V., Head J. F. & Akey C. W. The crystal structure of Drosophila NLP-core provides insight into pentamer formation and histone binding. Structure 11, 175–186 (2003). 12575937 10.1016/s0969-2126(03)00007-8

[b11] Namboodiri V. M., Akey I. V., Schmidt-Zachmann M. S., Head J. F. & Akey C. W. The structure and function of Xenopus NO38-core, a histone chaperone in the nucleolus. Structure 12, 2149–2160, doi: 10.1016/j.str.2004.09.017 (2004). 15576029

[b12] Frehlick L. J., Eirin-Lopez J. M. & Ausio J. New insights into the nucleophosmin/nucleoplasmin family of nuclear chaperones. Bioessays 29, 49–59, doi: 10.1002/bies.20512 (2007). 17187372

[b13] Hierro A. . Structural and functional properties of Escherichia coli-derived nucleoplasmin. A comparative study of recombinant and natural proteins. Eur J Biochem/FEBS 268, 1739–1748 (2001). 11248694

[b14] Dingwall C. . Nucleoplasmin cDNA sequence reveals polyglutamic acid tracts and a cluster of sequences homologous to putative nuclear localization signals. EMBO J 6, 69–74 (1987). 2884102 10.1002/j.1460-2075.1987.tb04720.x PMC553358

[b15] Prado A., Ramos I., Frehlick L. J., Muga A. & Ausio J. Nucleoplasmin: a nuclear chaperone. Biochem Cell Biol 82, 437–445, doi: 10.1139/o04-042 (2004). 15284896

[b16] Cotten M., Sealey L. & Chalkley R. Masive phosphorylation distinguishes Xenopus laevis nucleoplasmin isolated from oocytes and unfwertilized eggs. Biochemistry 25, 5063- 5069 (1986). 3768332 10.1021/bi00366a014

[b17] Ramos I., Prado A., Finn R. M., Muga A. & Ausio J. Nucleoplasmin-mediated unfolding of chromatin involves the displacement of linker-associated chromatin proteins. Biochemistry 44, 8274–8281, doi: 10.1021/bi050386w (2005). 15938617

[b18] Taneva S. G. . A mechanism for histone chaperoning activity of nucleoplasmin: thermodynamic and structural models. J Mol Biol 393, 448–463, doi: 10.1016/j.jmb.2009.08.005 (2009). 19683001

[b19] Ramos I. . Nucleoplasmin binds histone H2A–H2B dimers through its distal face. J Biol Chem 285, 33771–33778, doi: 10.1074/jbc.M110.150664 (2010). 20696766 PMC2962476

[b20] Ramos I. . The intrinsically disordered distal face of nucleoplasmin recognizes distinct oligomerization states of histones. Nucleic Acids Res 42, 1311–1325, doi: 10.1093/nar/gkt899 (2014). 24121686 PMC3902905

[b21] Stuwe T. . The FACT Spt16 “peptidase” domain is a histone H3-H4 binding module. Proc Natl Acad Sci USA 105, 8884–8889, doi: 10.1073/pnas.0712293105 (2008). 18579787 PMC2449335

[b22] Selth L. A. . An rtt109-independent role for vps75 in transcription-associated nucleosome dynamics. Mol Cel Biol 29, 4220–4234, doi: 10.1128/MCB.01882-08 (2009). PMC2715805 19470761

[b23] Finn R. M., Ellard K., Eirin-Lopez J. M. & Ausio J. Vertebrate nucleoplasmin and NASP: egg histone storage proteins with multiple chaperone activities. FASEB J 26, 4788–4804, doi: 10.1096/fj.12-216663 (2012). 22968912

[b24] Dimitrov S. & Wolffe A. P. Remodeling somatic nuclei in Xenopus laevis egg extracts: molecular mechanisms for the selective release of histones H1 and H1(0) from chromatin and the acquisition of transcriptional competence. EMBO J 15, 5897–5906 (1996). 8918467 PMC452350

[b25] Andrews A. J., Downing G., Brown K., Park Y. J. & Luger K. A thermodynamic model for Nap1-histone interactions. J Biol Chem 283, 32412–32418, doi: 10.1074/jbc.M805918200 (2008). 18728017 PMC2583301

[b26] Philpott A., Krude T. & Laskey R. A. Nuclear chaperones. Semin Cell Dev Biol 11, 7–14, doi: 10.1006/scdb.1999.0346 (2000). 10736259

[b27] Banks D. D. & Gloss L. M. Folding mechanism of the (H3-H4)2 histone tetramer of the core nucleosome. Protein Sci 13, 1304–1316, doi: 10.1110/ps.03535504 (2004). 15096635 PMC2286770

[b28] Bowman A. & Owen-Hughes T. Sulfyhydryl-reactive site-directed cross-linking as a method for probing the tetrameric structure of histones H3 and H4. Methods Mol Biol 833, 373–387, doi: 10.1007/978-1-61779-477-3_22 (2012). 22183605

[b29] Winkler D. D., Luger K. & Hieb A. R. Quantifying chromatin-associated interactions: the HI-FI system. Methods Enzymol 512, 243–274, doi: 10.1016/B978-0-12-391940-3.00011-1 (2012). 22910210

[b30] Liu W. H., Roemer S. C., Port A. M. & Churchill M. E. CAF-1-induced oligomerization of histones H3/H4 and mutually exclusive interactions with Asf1 guide H3/H4 transitions among histone chaperones and DNA. Nucleic Acids Res 40, 11229–11239, doi: 10.1093/nar/gks906 (2012). 23034810 PMC3526290

[b31] Bowman A., Ward R., El-Mkami H., Owen-Hughes T. & Norman D. G. Probing the (H3-H4)2 histone tetramer structure using pulsed EPR spectroscopy combined with site-directed spin labelling. Nucleic Acids Res 38, 695–707, doi: 10.1093/nar/gkp1003 (2010). 19914933 PMC2810997

[b32] Donham D. C., Scorgie J. K. & Churchill M. E. The activity of the histone chaperone yeast Asf1 in the assembly and disassembly of histone H3/H4-DNA complexes. Acids Res 39, 5449–5458, doi: 10.1093/nar/gkr097 (2011). PMC3141235 21447559

[b33] Mosammaparast N., Ewart C. S. & Pemberton L. F. A role for nucleosome assembly protein 1 in the nuclear transport of histones H2A and H2B. EMBO J 21, 6527–6538 (2002). 12456659 10.1093/emboj/cdf647 PMC136951

[b34] Andrews A. J., Chen X., Zevin A., Stargell L. A. & Luger K. The histone chaperone Nap1 promotes nucleosome assembly by eliminating nonnucleosomal histone DNA interactions. Mol Cell 37, 834–842, doi: 10.1016/j.molcel.2010.01.037 (2010). 20347425 PMC2880918

[b35] D’Arcy S. . Chaperone Nap1 shields histone surfaces used in a nucleosome and can put H2A–H2B in an unconventional tetrameric form. Mol Cell 51, 662–677, doi: 10.1016/j.molcel.2013.07.015 (2013). 23973327 PMC3878309

[b36] Winkler D. D., Zhou H., Dar M. A., Zhang Z. & Luger K. Yeast CAF-1 assembles histone (H3-H4)2 tetramers prior to DNA deposition. Acids Res 40, 10139–10149, doi: 10.1093/nar/gks812 (2012). PMC3488248 22941638

[b37] Winkler D. D., Muthurajan U. M., Hieb A. R. & Luger K. Histone chaperone FACT coordinates nucleosome interaction through multiple synergistic binding events. J Biol Chem 286, 41883–41892, doi: 10.1074/jbc.M111.301465 (2011). 21969370 PMC3308894

[b38] Kleinschmidt J. A., Fortkamp E., Krohne G., Zentgraf H. & Franke W. W. Co-existence of two different types of soluble histone complexes in nuclei of Xenopus laevis oocytes. J Biol Chem 260, 1166–1176 (1985). 2981836

[b39] Arnan C., Saperas N., Prieto C., Chiva M. & Ausio J. Interaction of nucleoplasmin with core histones. J Biol Chem 278, 31319–31324, doi: 10.1074/jbc.M305560200 (2003). 12791680

[b40] Banuelos S. . Activation mechanism of the nuclear chaperone nucleoplasmin: role of the core domain. J Mol Biol 334, 585–593 (2003). 14623196 10.1016/j.jmb.2003.09.067

[b41] Onikubo T. . Developmentally Regulated Post-translational Modification of Nucleoplasmin Controls Histone Sequestration and Deposition. Cell Rep, doi: 10.1016/j.celrep.2015.02.038 (2015). PMC4567554 25772360

[b42] Banuelos S. . Phosphorylation of both nucleoplasmin domains is required for activation of its chromatin decondensation activity. J Biol Chem 282, 21213–21221, doi: 10.1074/jbc.M702842200 (2007). 17510054

[b43] Hierro A., Arizmendi J. M., Banuelos S., Prado A. & Muga A. Electrostatic interactions at the C-terminal domain of nucleoplasmin modulate its chromatin decondensation activity. Biochemistry 41, 6408–6413 (2002). 12009903 10.1021/bi020002r

[b44] Korolev N., Vorontsova O. V. & Nordenskiold L. Physicochemical analysis of electrostatic foundation for DNA-protein interactions in chromatin transformations. Prog Biophys Mol Biol 95, 23–49, doi: 10.1016/j.pbiomolbio.2006.11.003 (2007). 17291569

[b45] Bohm V. . Nucleosome accessibility governed by the dimer/tetramer interface. Nucleic Acids Res 39, 3093–3102, doi: 10.1093/nar/gkq1279 (2011). 21177647 PMC3082900

[b46] Hieb A. R., D’Arcy S., Kramer M. A., White A. E. & Luger K. Fluorescence strategies for high-throughput quantification of protein interactions. Nucleic Acids Res 40, e33, doi: 10.1093/nar/gkr1045 (2012). 22121211 PMC3299996

[b47] Shintomi K., Takahashi T. S. & Hirano T. Reconstitution of mitotic chromatids with a minimum set of purified factors. Nat Cell Biol, doi: 10.1038/ncb3187 (2015). 26075356

[b48] Clement C. & Almouzni G. MCM2 binding to histones H3-H4 and ASF1 supports a tetramer-to-dimer model for histone inheritance at the replication fork. Nat Struct Mol Biol 22, 587–589, doi: 10.1038/nsmb.3067 (2015). 26243657

[b49] Kimura H. & Cook P. R. Kinetics of core histones in living human cells: little exchange of H3 and H4 and some rapid exchange of H2B. J Cell Biol 153, 1341–1353 (2001). 11425866 10.1083/jcb.153.7.1341 PMC2150718

[b50] Thiriet C. & Hayes J. J. Replication-independent core histone dynamics at transcriptionally active loci *in vivo*. Genes Dev 19, 677–682, doi: 10.1101/gad.1265205 (2005). 15769942 PMC1065721

[b51] Dion M. F. . Dynamics of replication-independent histone turnover in budding yeast. Science 315, 1405–1408, doi: 10.1126/science.1134053 (2007). 17347438

[b52] Annunziato A. T. Split decision: what happens to nucleosomes during DNA replication? J Biol Chem 280, 12065–12068, doi: 10.1074/jbc.R400039200 (2005). 15664979

[b53] Xu M. . Partitioning of histone H3-H4 tetramers during DNA replication-dependent chromatin assembly. Science 328, 94–98, doi: 10.1126/science.1178994 (2010). 20360108

[b54] Katan-Khaykovich Y. & Struhl K. Splitting of H3-H4 tetramers at transcriptionally active genes undergoing dynamic histone exchange. Proc Natl Acad Sci USA 108, 1296–1301, doi: 10.1073/pnas.1018308108 (2011). 21220302 PMC3029712

[b55] Berndsen C. E. . Molecular functions of the histone acetyltransferase chaperone complex Rtt109-Vps75. Nat Struct Mol Biol 15, 948–956 (2008). 19172748 10.1038/nsmb.1459 PMC2678805

[b56] Park Y. J. & Luger K. Histone chaperones in nucleosome eviction and histone exchange. Curr Opin Struct Biol 18, 282–289, doi: 10.1016/j.sbi.2008.04.003 (2008). 18534842 PMC2525571

[b57] Tsubota T. . Histone H3-K56 acetylation is catalyzed by histone chaperone-dependent complexes. Mol Cell 25, 703–712, doi: 10.1016/j.molcel.2007.02.006 (2007). 17320445 PMC1853276

[b58] Bowman A. . The histone chaperones Nap1 and Vps75 bind histones H3 and H4 in a tetrameric conformation. Mol Cell 41, 398–408, doi: 10.1016/j.molcel.2011.01.025 (2011). 21329878 PMC3093613

[b59] Chen S. . Structure-function studies of histone H3/H4 tetramer maintenance during transcription by chaperone Spt2. Genes Dev 29, 1326–1340, doi: 10.1101/gad.261115.115 (2015). 26109053 PMC4495402

[b60] Ausio J., Dong F. & van Holde K. E. Use of selectively trypsinized nucleosome core particles to analyze the role of the histone “tails” in the stabilization of the nucleosome. J Mol Biol 206, 451–463 (1989). 2716057 10.1016/0022-2836(89)90493-2

[b61] Garcia-Ramirez M., Leuba S. H. & Ausio J. One-step fractionation method for isolating H1 histones from chromatin under nondenaturing conditions. Protein Expr Purif 1, 40–44 (1990). 2152183 10.1016/1046-5928(90)90043-x

[b62] Dyer P. N. . Reconstitution of nucleosome core particles from recombinant histones and DNA. Methods Enzymol 375, 23–44 (2004). 14870657 10.1016/s0076-6879(03)75002-2

[b63] Park Y. J., Dyer P. N., Tremethick D. J. & Luger K. A new fluorescence resonance energy transfer approach demonstrates that the histone variant H2AZ stabilizes the histone octamer within the nucleosome. J Biol Chem 279, 24274–24282, doi: 10.1074/jbc.M313152200 (2004). 15020582

[b64] Cheng Y.-C. P. & Mouse W. H. ascites sarcoma 180 Thymidilate kinase. General properties, kinetic analysis, and inhibition studies. Biochemistry 12, 2612–2619 (1973). 4711469 10.1021/bi00738a010

