# Supplementary material for: A Quantitative Characterization of Nucleoplasmin/Histone Complexes Reveals Chaperone Versatility
Source: Sci Rep. 2016 Aug 25;6:32114. doi: 10.1038/srep32114 (PMC4997359; doi:10.1038/srep32114)
Supplement: Supplementary Information [file srep32114-s1.pdf]

SUPPLEMENTARY INFORMATION

A QUANTITATIVE CHARACTERIZATION OF NUCLEOPLASMIN/HISTONE COMPLEXES REVEALS CHAPERONE VERSATILITY

Noelia Fernández-Rivero, Aitor Franco, Adrian Velazquez-Campoy, Edurne Alonso, Arturo Muga, and Adelina Prado

Supplementary Materials and Methods

NP-mediated sperm chromatin decondensation and nucleosome assembly. Demembranated sperm nuclei from *Xenopus laevis* were prepared as reported previously[1]. To prepare demembranated sperm nuclei, frogs were anesthetized with MS-222 and sacrificed according to standard and approved protocols (University of the Basque Country (UPV/EHU), Institutional Animal Care Committee with guidelines from the Spanish Council on Animal Care) (CEBA/51- PO3-03/2010)). The nuclear density (nuclei per ml) of the sperm was estimated from the total DNA nuclear content determined from the absorbance at 260 nm of a nuclear suspension lysed in the presence of SDS[2]. A relationship of 3×10^4 nuclei per 0.14 μg of DNA was used[3]. 8 μg DNA nuclei were incubated with NP/H2A-H2B complexes (1/0.5 molar ratio; 3.5 μM NP final concentration) obtained with the different native and recombinant NP variants. The buffer was 150 mM KCl, 25 mM Tris-HCl, pH 7.5, 2 mM $MgCl_2$ (buffer 1). As a control, the same experiment was done in the absence of NP.

Electrophoretic analysis of NP-mediated sperm chromatin decondensation and nucleosome assembly. AUT-PAGE (5% acetic acid, 2.5 M urea, 6 mM Triton X-100, 15% PAGE) was used to characterize the chromosomal proteins removed from sperm chromatin by NP/H2A-H2B (1/0.5 molar ratio) complexes. Briefly, the corresponding sperm (8 μg per assay) were incubated in buffer 1, 100 μl final volume, as described above. Upon incubation at room temperature for 1 h, the nuclear suspension was centrifuged (16000 x g for 30 min at 4ºC). The resulting supernatants contain NP and basic proteins associated with NP, and the pellets contain the basic proteins that remain associated to DNA. The supernatants and pellets were treated as described[4] and proteins were analysed by AUT-PAGE. The analysis of the chromatin structure using microccocal nuclease was performed essentially as described before[5]. Briefly, sperm nuclei (8 μg) in buffer 1 were incubated 1 h with preformed NP/H2A-H2B (1/0.5 molar

ratio) complexes (3.5 μM NP). Then, 1 mM $CaCl_2$ was added and DNA was digested with 0.5 U of microccocal nuclease (Sigma) during 5 min at room temperature. The reaction was stopped with 10 mM EDTA and digested with 25 μg proteinase K. Purified DNA was resolved in 1.5% agarose native gel.

Electrophoresis Mobility Shift Assay (EMSA). eNP (2 μM) was mixed with different histone concentrations and incubated in 150 mM NaCl, 20 mM Tris-HCl, pH 7.6, 1 mM DTT at 25ºC for 1 h. Native-PAGE was carried out in NativePAGE™ Novex™ 4-16% Bis-Tris Gels (Invitrogen), and proteins were stained with Coomassie Brilliant Blue.

Supplementary Figure Legends

Supplementary Figure 1. Fluorescence studies of the interaction of core histones with eNP. A) Emission spectra of H2A-H2BT112C-Alexa 488 in the absence (black) and presence of eNP (gray) at 0.15 M NaCl (solid line) or 2 M NaCl (dashed line). eNP/histone molar ratio was 50/1. **B**) Same as in A for dimeric H3C110E-H4T71C-Alexa 488 at an eNP/histone molar ratio of 50/1. **C**) Same as in A and B for tetrameric cross-linked H3C110AK115C-H4T71C-Alexa 488 at an eNP/histone molar ratio of 1/2. The spectra of the histone (black dotted) and the eNP/histone complex (gray dotted) in the presence of 3 M GdnHCl are also shown.

Supplementary Figure 2. NP-mediated dissociation of basic proteins from DNA and nucleosome assembly. A, B) Demembranated sperm nuclei in buffer 1 were incubated with different NP/H2A-H2B (1/0.5 molar ratio) complexes or histones alone (control). Samples were centrifuged and the supernatants (A) and pellets (B) analysed by AUT-PAGE as described in Supplementary Materials and Methods. **C**) Sperm chromatin structure after incubation with NP/H2A-H2B (1/0.5 molar ratio) complexes, analysed by 1.5% agarose gel electrophoresis after micrococcal nuclease digestion. Lane CR contains linker histone-depleted chromatin.

Supplementary Figure 3. Formation of eNP/ H3C110A-H4T71C and eNP/H3C110AK115C-H4T71C complexes as seen by Native-PAGE. Complex formation between eNP (2μM pentamer) and recombinant H3C110A-H4T71C (A) or cross-linked H3C110AK115C-H4T71C (B).

Supplementary References

1 Lohka, M. J. Analysis of nuclear envelope assembly using extracts of Xenopus eggs. *Methods Cell Biol* **53**, 367-395 (1998).

2 Wang, X., Moore, S. C., Laszckzak, M. & Ausio, J. Acetylation increases the alpha-helical content of the histone tails of the nucleosome. *J Biol Chem* **275**, 35013-35020, doi:10.1074/jbc.M004998200 (2000).

3 Rice, P., Garduno, R., Itoh, T., Katagiri, C. & Ausio, J. Nucleoplasmin-mediated decondensation of Mytilus sperm chromatin. Identification and partial characterization of a nucleoplasmin-like protein with sperm-nuclei decondensing activity in Mytilus californianus. *Biochemistry* **34**, 7563-7568 (1995).

4 Ramos, I., Prado, A., Finn, R. M., Muga, A. & Ausio, J. Nucleoplasmin-mediated unfolding of chromatin involves the displacement of linker-associated chromatin proteins. *Biochemistry* **44**, 8274-8281, doi:10.1021/bi050386w (2005).

5 Hierro, A., Arizmendi, J. M., Banuelos, S., Prado, A. & Muga, A. Electrostatic interactions at the C-terminal domain of nucleoplasmin modulate its chromatin decondensation activity. *Biochemistry* **41**, 6408-6413 (2002).

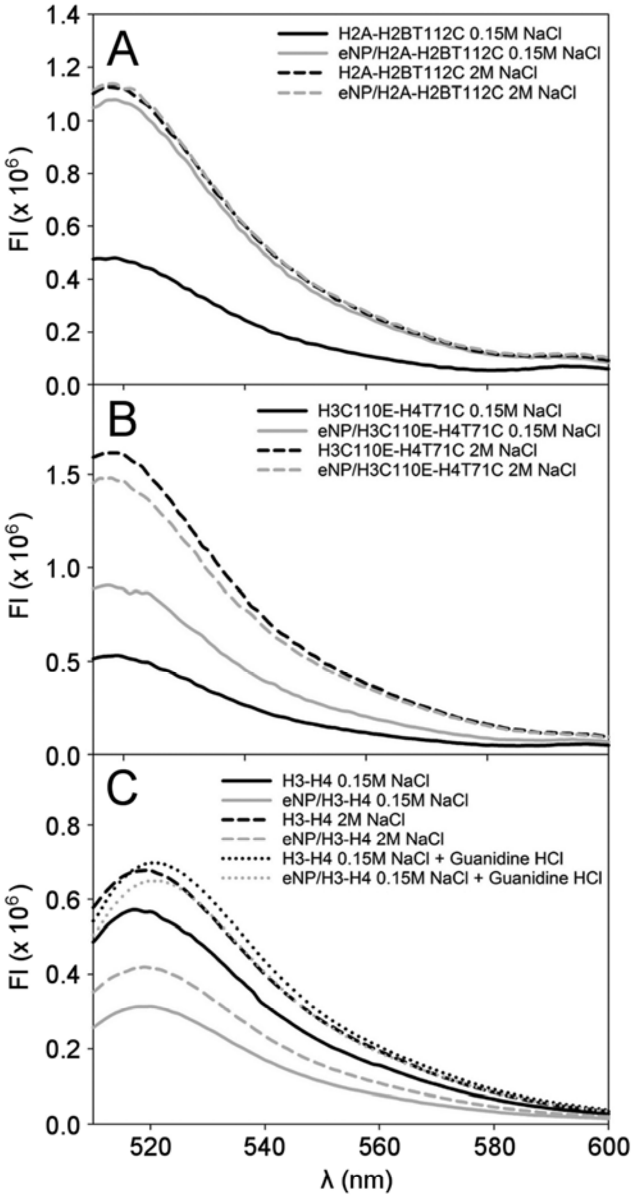
A
H2A-H2BT112C 0.15M NaCl
eNP/H2A-H2BT112C 0.15M NaCl
H2A-H2BT112C 2M NaCl
eNP/H2A-H2BT112C 2M NaCl
B
H3C110E-H4T71C 0.15M NaCl
eNP/H3C110E-H4T71C 0.15M NaCl
H3C110E-H4T71C 2M NaCl
eNP/H3C110E-H4T71C 2M NaCl
C
H3-H4 0.15M NaCl
eNP/H3-H4 0.15M NaCl
H3-H4 2M NaCl
eNP/H3-H4 2M NaCl
H3-H4 0.15M NaCl + Guanidine HCl
eNP/H3-H4 0.15M NaCl + Guanidine HCl
FI (x 10^6)
λ (nm)
520
540
560
580
600

FIGURE S1

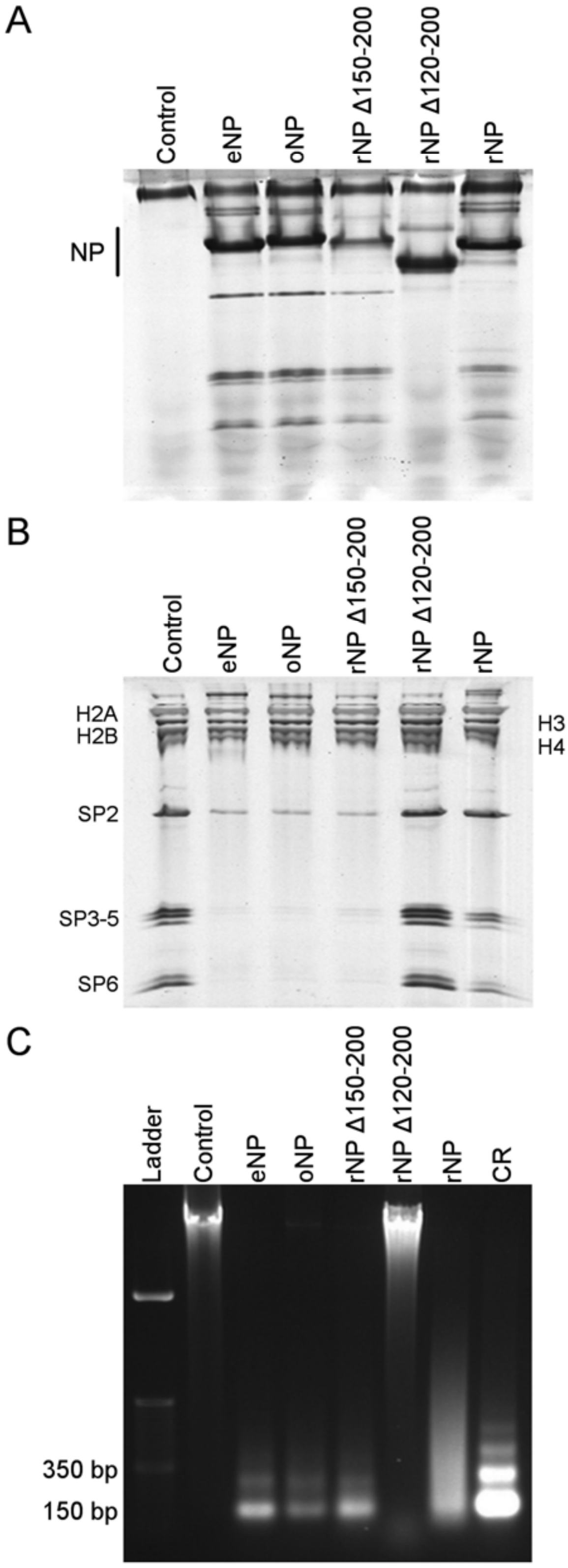
A
Control
eNP
oNP
rNP Δ150-200
rNP Δ120-200
rNP
NP
B
Control
eNP
oNP
rNP Δ150-200
rNP Δ120-200
rNP
H2A
H2B
H3
H4
SP2
SP3-5
SP6
C
Ladder
Control
eNP
oNP
rNP Δ150-200
rNP Δ120-200
rNP
CR
350 bp
150 bp

FIGURE S2

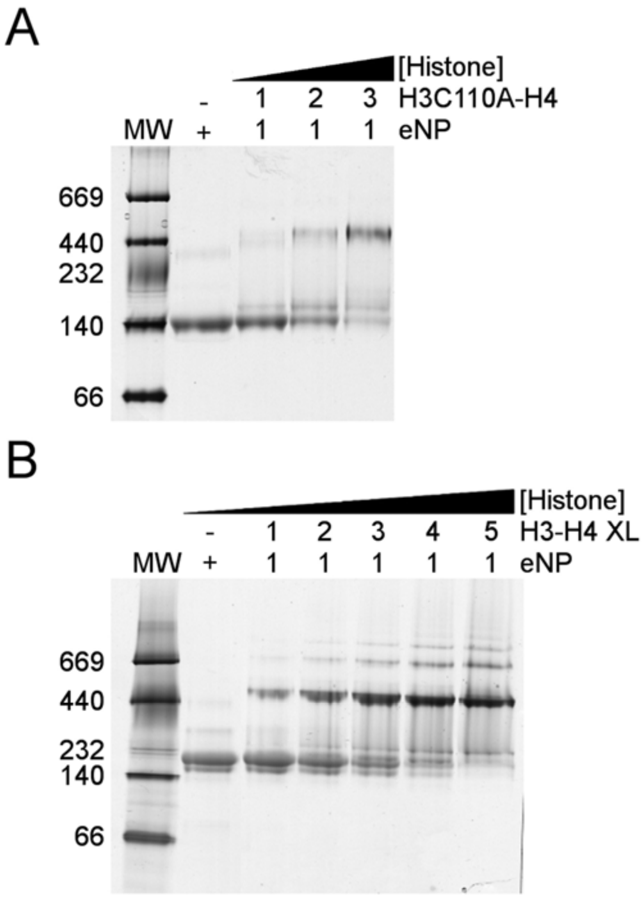
A
[Histone]
- 1 2 3 H3C110A-H4
MW + 1 1 1 eNP
669
440
232
140
66
B
[Histone]
- 1 2 3 4 5 H3-H4 XL
MW + 1 1 1 1 1 eNP
669
440
232
140
66

FIGURE S3
